# Utility of Coronary Computed Tomography Angiography in Patients Undergoing Transcatheter Aortic Valve Implantation: A Meta-Analysis and Meta-Regression Based on Published Data from 7458 Patients

**DOI:** 10.3390/jcm13020631

**Published:** 2024-01-22

**Authors:** Gerhard-Paul Diller, Mirjam Gerwing, Simona Boroni Grazioli, Fernando De-Torres-Alba, Robert M. Radke, Julia Vormbrock, Helmut Baumgartner, Gerrit Kaleschke, Stefan Orwat

**Affiliations:** 1 Department of Cardiology III, Adult Congenital and Valvular Heart Disease, University Hospital Muenster, 48149 Muenster, Germanygerrit.kaleschke@ukmuenster.de (G.K.); orwat@ukmuenster.de (S.O.); 2 Clinic of Radiology, University Hospital Muenster, 48149 Muenster, Germany

**Keywords:** meta-analysis, coronary CT angiography, transcatheter aortic valve implantation, coronary artery disease, diagnostic accuracy

## Abstract

Background: Coronary CT angiography (CCTA) may detect coronary artery disease (CAD) in transcatheter aortic valve implantation (TAVI) patients and may obviate invasive coronary angiography (ICA) in selected patients. We assessed the diagnostic accuracy of CCTA for detecting CAD in TAVI patients based on published data. Methods: Meta-analysis and meta-regression were performed based on a comprehensive electronic search, including relevant studies assessing the diagnostic accuracy of CCTA in the setting of TAVI patients compared to ICA. The sensitivity, specificity, positive predictive value (PPV), and negative predictive value (NPV), were calculated on a patient and per segment level. Results: Overall, 27 studies (total of 7458 patients) were included. On the patient level, the CCTA’s pooled sensitivity and NPV were 95% (95% CI: 93–97%) and 97% (95% CI: 95–98%), respectively, while the specificity and PPV were at 73% (95% CI: 62–82%) and 64% (95% CI: 57–71%), respectively. On the segmental coronary vessel level, the sensitivity and NPV were 90% (95% CI: 79–96%) and 98% (95% CI: 97–99%). Conclusions: This meta-analysis highlights CCTA’s potential as a first-line diagnostic tool although its limited PPV and specificity may pose challenges when interpreting heavily calcified arteries. This study underscores the need for further research and protocol standardization in this area.

## 1. Introduction

Aortic stenosis is one of the leading valvular conditions, globally, and is associated with significant morbidity and mortality, especially in elderly patients [1,2,3,4]. Transcatheter aortic valve implantation (TAVI) offers a less invasive alternative to traditional surgical valve replacement, especially in medium- and high-risk patients [5,6,7]. The presence of concomitant obstructive coronary artery disease (CAD) can complicate TAVI, thus necessitating appropriate pre-procedural evaluation to optimize patient outcomes [8].

Computed tomography (CT) scans are essential in the pre-procedural planning for TAVI in patients with aortic stenosis. Current clinical practice, supported by the literature and guideline recommendations, includes the use of a pre-TAVI CT scan for valve assessment and technical procedure planning [9]. Specifically, CT allows for detailed ana-tomic valve visualization and quantification of the grade of calcification. The degree and distribution of calcification can guide prosthetic valve choice and predict the risk of complications, such as pacemaker dependency and paravalvular leaks [10,11,12,13]. Accurate measurements of the aortic annulus, leaflets, and sinotubular junction are also critical for selecting appropriately sized prosthetic valves. In selected patients, CT may also allow for the identification of bicuspid aortic valve disease, which may be associated with an abnormal morphology and may require different procedural strategies. Pre-TAVI CT assessment of the aorta, up to the level of the femoral artery distally, is essential for vascular access pathway assessment [14]. Additionally, CT angiography assesses the caliber, tortuosity, and calcification of potential access routes (transfemoral, transapical, or transaortic), enabling clinicians to determine the most suitable access pathway [9]. Regarding coronary artery evaluation, CT assessment is established for the delineation of the coronary ostia and their height and location relative to the aortic annulus. This information is essential for pre-procedure planning and for preventing the obstruction of coronary arteries during valve deployment [15]. Coronary CT angiography (CCTA) can further identify significant coronary artery disease (CAD) requiring percutaneous coronary intervention. The uptake of CCTA-based CAD assessment and, especially, the partial replacement of invasive coronary angiography (ICA) by CCTA before TAVI has been slow in clinical practice. To date, ICA remains the gold standard for diagnosing CAD in this setting. However, ICA carries small but non-negligible risks, such as bleeding and vascular complications [16,17]. These are often exacerbated by repeated puncture of the femoral artery, which can complicate the TAVI procedure itself. Given these aspects, a shift towards non-invasive diagnostic modalities with CCTA at the forefront has been advocated [18,19]. CCTA, with its high spatial resolution and its ability to visualize coronary artery lumens and atherosclerotic plaques, offers a promising alternative. Despite the potential advantages of CCTA, its accuracy in detecting obstructive CAD in patients referred for TAVI remains a subject of debate. This population often presents with heavily calcified arteries, which can pose challenges during CCTA interpretation and potentially lead to diagnostic inaccuracies [20].

The current meta-analysis aims to synthesize the existing literature on the diagnostic performance of CCTA in this specific clinical scenario. Our analysis extends the data provided by previous meta-analytic approaches in this setting, [18,19,21] by utilizing a larger number of patients, including contemporary studies (including novel technologies, such as photon counting CTs), pooling the results on the segmental, graft, and proximal artery levels and utilizing a meta-regression approach to assess possible modifiers of diagnostic accuracy. The primary objective of the meta-analysis was to evaluate the sensitivity, specificity, positive predictive value, and negative predictive value of CCTA in detecting obstructive CAD on a patient and coronary artery segment level, among patients referred for TAVI. Secondary objectives include assessing the temporal aspects of coronary artery disease and the association between patient characteristics and the diagnostic accuracy of CCTA in this setting, based on meta-regression approaches.

## 2. Methods

### 2.1. Study Selection

**Figure 1:**
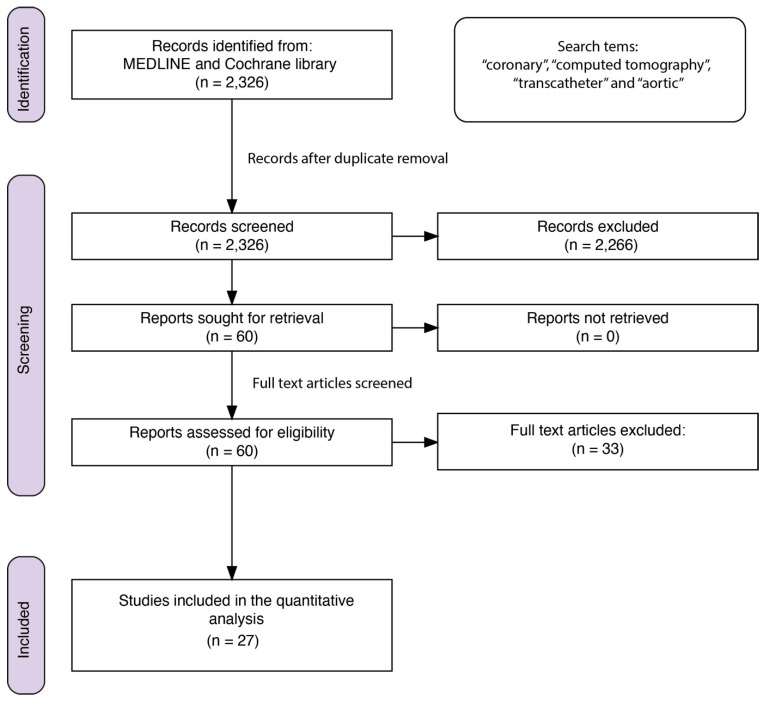
PRISMA flow diagram.

We conducted a comprehensive search on the MEDLINE and Cochrane Library databases to identify relevant primary studies assessing the diagnostic accuracy of CCTA in the setting of TAVI patients (date of search 14 November 2023). The search terms employed included the terms “coronary”, “computed tomography”, “transcatheter”, and “aortic”. Figure 1 illustrates the PRISMA flow diagram of the screening strategy [22]. To avoid missing relevant studies a broader search strategy was also employed, querying databases for the terms (“cardiac” AND “multidetector” AND “CT”), as well as (“aortic” AND “multidetector” AND “CT”) or (“aortic” AND “stenosis” AND “CT”). This resulted in 2295, 1278, and 2369 results, respectively. After removing duplicates, 5044 articles remained. The titles and abstracts of these manuscripts were screened manually (GD and SO); however, no additional relevant manuscripts could be identified for inclusion in the quantitative analysis. This allowed for a broad search, maximizing the yield, while accepting the fact that numerous documents had to be screened and excluded manually. No publication year time limits were included in our search strategies. Two investigators (GPD and SO) screened all the initial electronic matches manually, only including studies providing sufficient data depth suitable for a meta-analytic approach. The inclusion criteria were studies evaluating the diagnostic accuracy of CCTA in patients pre-TAVI in comparison to invasive coronary angiography (ICA). Studies providing sufficient data to construct 2 × 2 contingency tables for true positives (TPs), false positives (FPs), true negatives (TNs), and false negatives (FNs) were included. If the studies provided sufficient information to deduct this information from the total number of patients included and the calculated parameters, such as sensitivity, specificity, positive predictive value (PPV), and negative predictive value (NPV), the studies were also included. All the included publications are based on peer-reviewed articles published in English. The exclusion criteria were case reports, editorials, and review articles, as well as studies focusing on populations other than those undergoing TAVI. Studies not comparing pre-TAVI CCTA to ICA as the reference standard were also excluded. Two independent reviewers (GPD and SO) extracted data from the selected studies. The extracted information included study characteristics (author, year of publication, study design), patient demographics, CCT protocols, and diagnostic accuracy measures. In addition, we collected information on gender distribution, relevant baseline demographics, cardiovascular risk factors, CAD, previous coronary percutaneous or surgical coronary procedures, atrial fibrillation, and details on the CCTA assessment. Consistent with the literature, relevant CAD was defined as the presence of ≥50% stenosis as the main criteria, while ≥70% diameter reduction was considered as an additional endpoint. Native, stented arteries, and coronary artery bypass grafts were also considered for analysis. Discrepancies between reviewers were resolved through discussion or consultation with a third co-author (SBG). The quality of the included studies was assessed using the quality assessment of diagnostic accuracy studies-2 (QUADAS-2) tool, evaluating the potential risk of bias and applicability concerns across four domains: patient selection, index test, reference standard, and flow and timing [23].

### 2.2. Statistical Analysis

Diagnostic accuracy measures, including sensitivity, specificity, positive predictive value (PPV), and negative predictive value (NPV), were calculated for each study. For studies reporting numbers, sensitivity, specificity, PPV and NPV only, the TPs, FPs, TNs, and FNs were derived by solving the relevant equations (mathematically representing 5 equations with 5 unknowns), using a custom written computer program. The relevant pooled estimates for sensitivity, specificity, PPV, and NPV were subsequently obtained using a random effects model and the heterogeneity among the studies was assessed using the I^2^ statistic. Subgroup analyses were conducted, based on relevant study characteristics, to explore potential sources of heterogeneity. The effect of the moderators was assessed using subgroup analyses (for nominal covariates) and by regressing the effect sizes of the meta-analytic model (for continuous variables) [24]. Estimates of the number of patients classified correctly, based on the diagnostic accuracy measures, were obtained based on the methods provided by the MetaDTA (Diagnostic Test Accuracy Meta-analysis) framework, as outlined previously [25]. Publication bias was evaluated using Deeks’ funnel plot asymmetry test. The overall diagnostic performance of CCTA was summarized using hierarchical summary receiver operating characteristic (hsROC) analysis and the pAUC values are provided [26]. A two-sided *p*-value of less than 0.05 was considered statistically significant. All statistical analyses were performed using R statistical software (version 4.3.1, The R Foundation for Statistical Computing), including the *meta* and *metafor* packages.

## 3. Results

**Figure 2:**
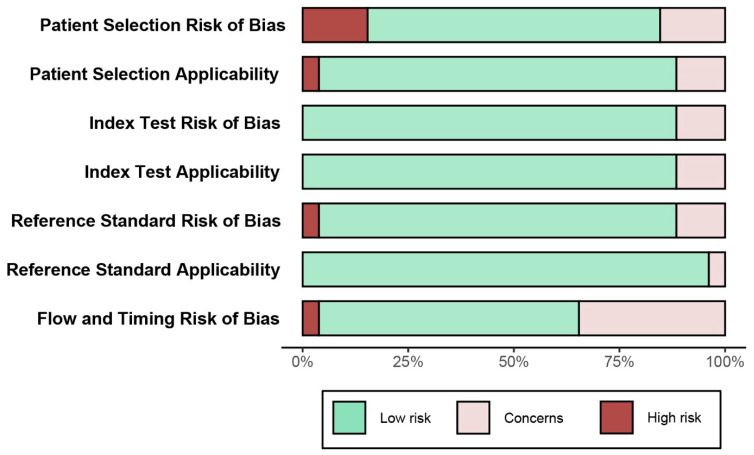
Results from the quality assessment of diagnostic accuracy studies-2 (QUADAS-2) analysis, illustrating a low overall risk of bias for the analysis cohort.

Overall, 2326 studies were retrieved and screened for inclusion in the current analysis. The titles and electronic abstracts of the studies were screened manually, and 60 papers were selected for further, complete review. The full-text versions of these manuscripts were manually reviewed, and 27 studies [27,28,29,30,31,32,33,34,35,36,37,38,39,40,41,42,43,44,45,46,47,48,49,50,51,52,53], fulfilling the inclusion criteria and providing appropriate data, were ultimately included. These studies were published between 2011 and 2023 and included 7458 patients. The results of the QUADAS-2 assessment are presented in Figure 2. While relevant heterogeneity in the inclusion and exclusion criteria (e.g., inclusion or exclusion of patients with previous coronary interventions or atrial fibrillation) existed between the studies, most studies included consecutive patients, thus reducing the risk of bias. No evidence of publication bias was detected using Deeks’ funnel plot asymmetry test, as shown in the Supplementary Materials. Table 1 provides an overview of the studies, including patient characteristics, scanner details, and the presence of previous coronary interventions or coronary artery bypass surgery. Descriptive details on the inclusion and exclusion criteria applicable to the included studies are listed in the Supplementary Materials.

### 3.1. Per Patient Analysis

**Figure 3:**
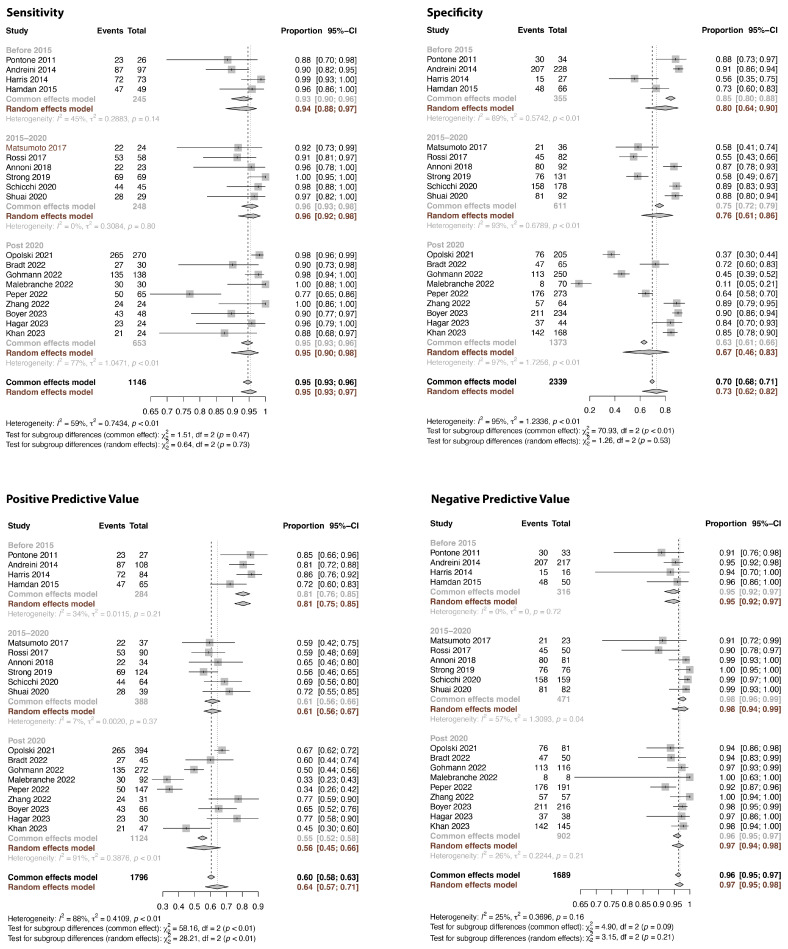
Sensitivity, specificity, positive and negative predictive value on the patient level, comparing coronary computed tomography with invasive angiography using a cut-off value of 50% lumen stenosis. Studies were stratified by year of publication [27,28,29,30,32,34,35,36,37,40,41,43,44,45,47,49,50,51,53].

**Figure 4:**
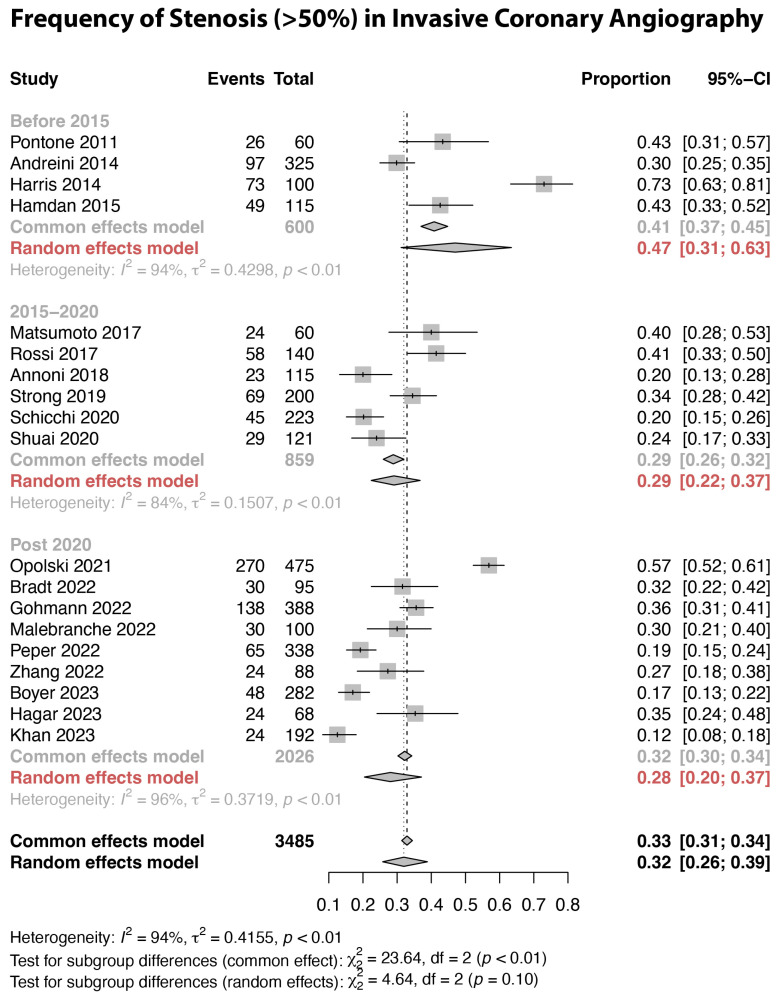
Frequency of at least 50% stenosis in invasive coronary angiography across the included studies. The frequency of ≥50% stenosis was calculated as the sum of true positives and false negatives provided [27,28,29,30,32,34,35,36,37,40,41,43,44,45,47,49,50,51,53].

**Figure 5:**
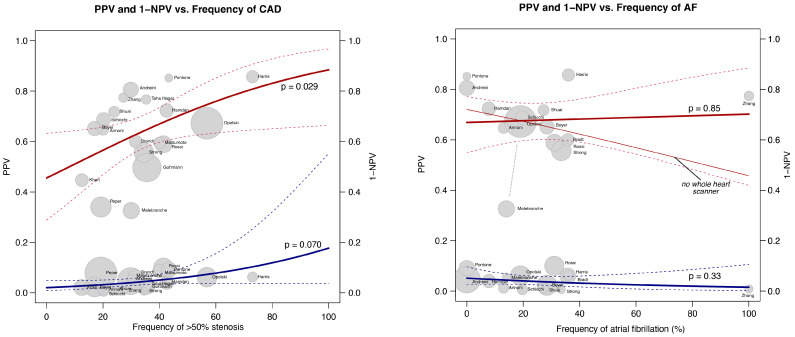
Association between frequency of coronary stenoses (>50%) or frequency of atrial fibrillation in the study cohort and the positive predictive value (PPV, red line) or 1—negative predictive value (NPV, blue line) in the studies based on the results of the meta-regression analysis. For details see text [27,28,29,30,32,34,35,36,37,40,41,43,44,45,47,49,50,51,53].

The results for sensitivity, specificity, PPV, and NPV on the patient level, comparing CCTA with ICA (using a cut-off value of 50% diameter reduction), are presented in Figure 3 and Table 2. The overall pooled values were 0.95 (95% CI: 0.93–0.97) for sensitivity, 0.73 (95% CI: 0.62–0.82) for specificity, 0.64 (95% CI: 0.57–0.71) for PPV, and 0.97 (95% CI: 0.95–0.98) for NPV, respectively. While heterogeneity of the results was evident for all measures, this was particularly evident for specificity and PPV. Inspecting the forest plot suggested a temporal trend for PPV, with lower values in the current era (post-2020) compared to the 2010–2015 period. This visual impression was confirmed using meta-regression analysis of the publication year vs. the PPV (*p* = 0.001). As the PPV is dependent on the prevalence of the condition in the study population, we investigated the temporal trend of the frequency of at least 50% stenosis on invasive coronary angiography across the included studies. The frequency of ICA derived ≥50% stenosis was calculated as the sum of true positives and false negatives provided in the studies. This showed that the frequency of ≥50% stenosis decreased from a pooled average of 47% before 2015 to 28% in the current era (Figure 4), and this was confirmed using meta-regression analysis (*p* = 0.028, average annual decrease of 2.3%). In fact, before 2015, none of the studies explicitly excluded PCI patients, while post-2020, 64.3% of studies did not include any patients with previous PCI. Accordingly, the pooled frequency of pre-CCTA PCI in the random effects models decreased from 28% before 2015 to 2% between 2015 and 2020 and <1% post-2020. Similarly, patients with previous CABG were less frequently included in the current era (<1%) compared to the pre-2015 period (21%). As illustrated in Table 3, assessing the association between the patient and the CT-related variables and parameters of diagnostic accuracy, using univariable meta-regression analysis, the occurrence of coronary artery stenosis was associated with PPV and to a lesser extent specificity. Figure 5 illustrates the associations between CAD frequency (≥50% stenosis) or the frequency of atrial fibrillation in the study and the PPV. 

**Figure 6:**
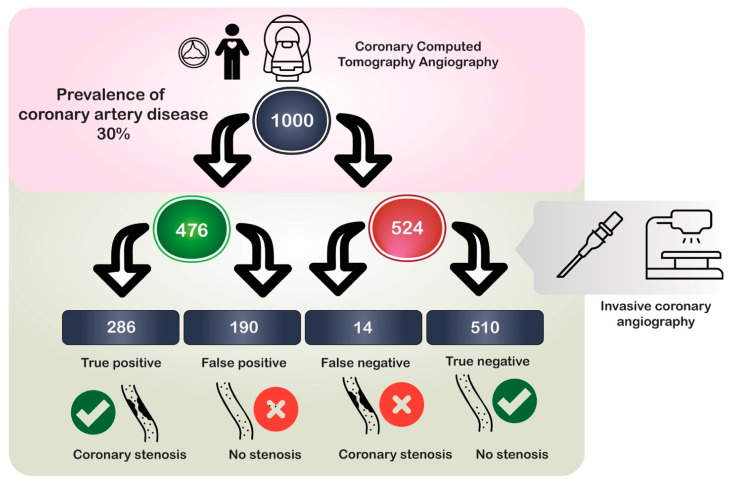
Illustration of the potential flow of patients based on the results of the current meta-analysis. Underlying data are based on patient-level data, comparing coronary computed tomography with invasive angiography, using a cut-off value of 50% lumen stenosis.

**Figure 7:**
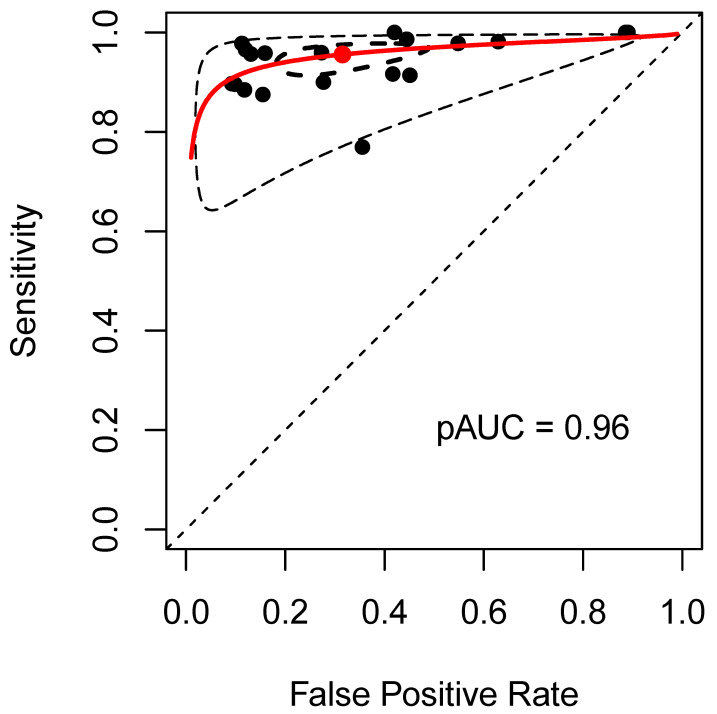
Summary receiver operating characteristic curve results for diagnostic test accuracy based on the patient-level bivariate meta-analysis, with a cut-off value of 50% luminal stenosis, comparing coronary computed tomography and invasive coronary angiography. Abbreviation: pAUC = partial area under the ROC curve.

We also estimated the proportion of patients diagnosed correctly using CCTA depending on the prevalence of CAD (≥50% stenosis) in the underlying population. Table 4 shows that the proportion of correctly diagnosed patients increases with the frequency of underlying CAD in the population. The potential flow of patients based on the results of the current meta-analysis is illustrated in Figure 6. Furthermore, the results of the summary receiver operating characteristic (sROC) curve (based on the bivariate model) for diagnostic test accuracy are presented in Figure 7. This analysis confirmed a good discriminatory ability of CCTA, with a pAUC value of 0.96.

Further insight into the diagnostic accuracy measures on the patient level, using a cut-off value of at least 70% luminal narrowing, is provided in Figure S1 (Supplementary Materials). The overall pooled values in the random effects model were 0.96 (95% CI: 0.85–0.99) for sensitivity, 0.78 (95% CI: 0.65–0.86) for specificity, 0.62 (95% CI: 0.49–0.73) for PPV, and 0.98 (95% CI: 0.94–0.99) for NPV, respectively.

### 3.2. Per Coronary Segment Analysis

This analysis is based on the coronary artery segment classification, using 50% luminal stenosis as an endpoint. In principle, two potential approaches are available to deal with unanalyzable segments. Firstly, analyses can be restricted to segments with adequate image quality, and diagnostic accuracy, reported specifically for these segments or, secondly, unanalyzable segments can be assumed to be stenosed and coded as such. 

**Figure 8:**
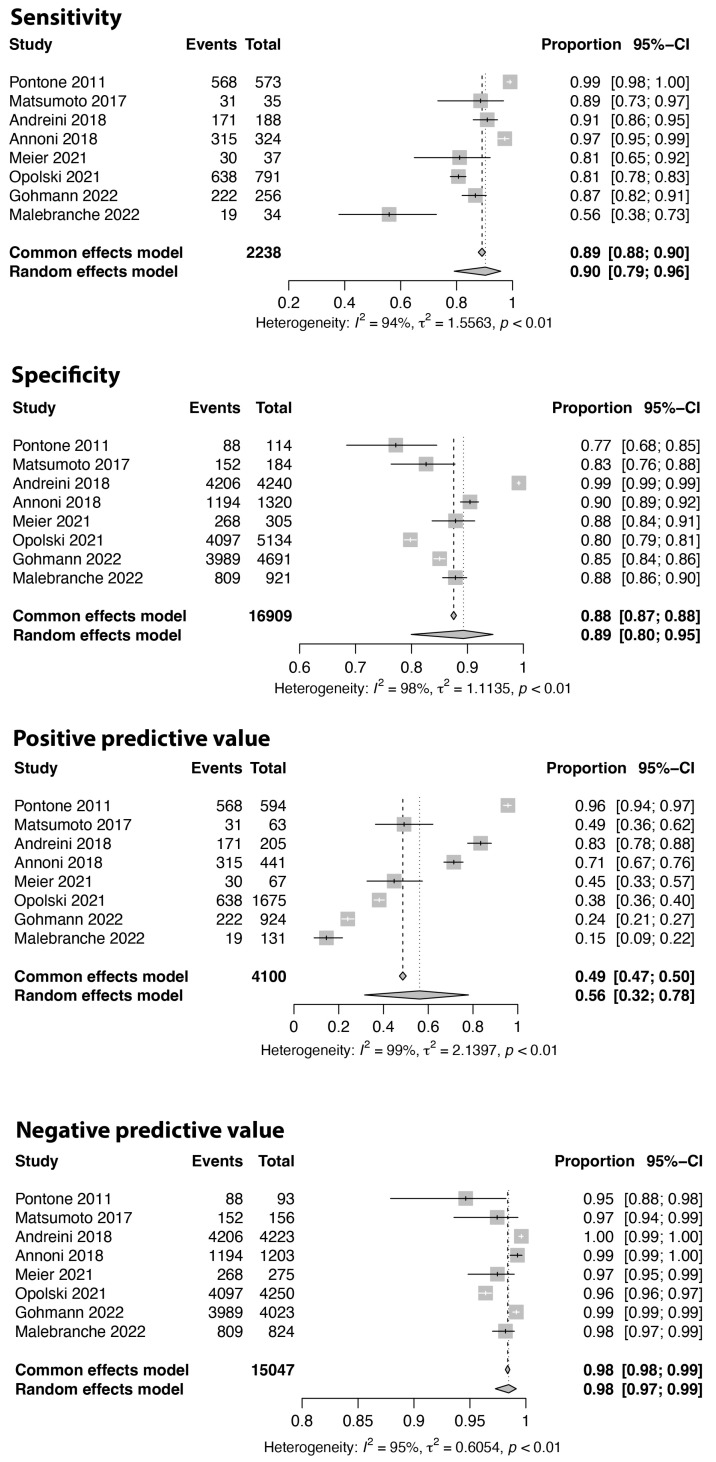
Sensitivity, specificity, positive and negative predictive values on the segment, coronary artery, level. Only evaluable segments are considered for analysis. Comparison between coronary computed tomography and invasive angiography, using a cut-off value of 50% lumen stenosis, are displayed [27,28,32,40,41,42,43,45].

Based on the analysis of eight studies, including 19,147 segments, the sensitivity, specificity, PPV, and NPV for the evaluable segments were 0.90 (95% CI: 0.79–0.96), 0.89 (95% CI: 0.80–0.95), 0.56 (95% CI: 0.32–0.78), and 0.98 (95% CI: 0.97–0.99) in the pooled analysis, respectively, as illustrated in Figure 8. 

**Figure 9:**
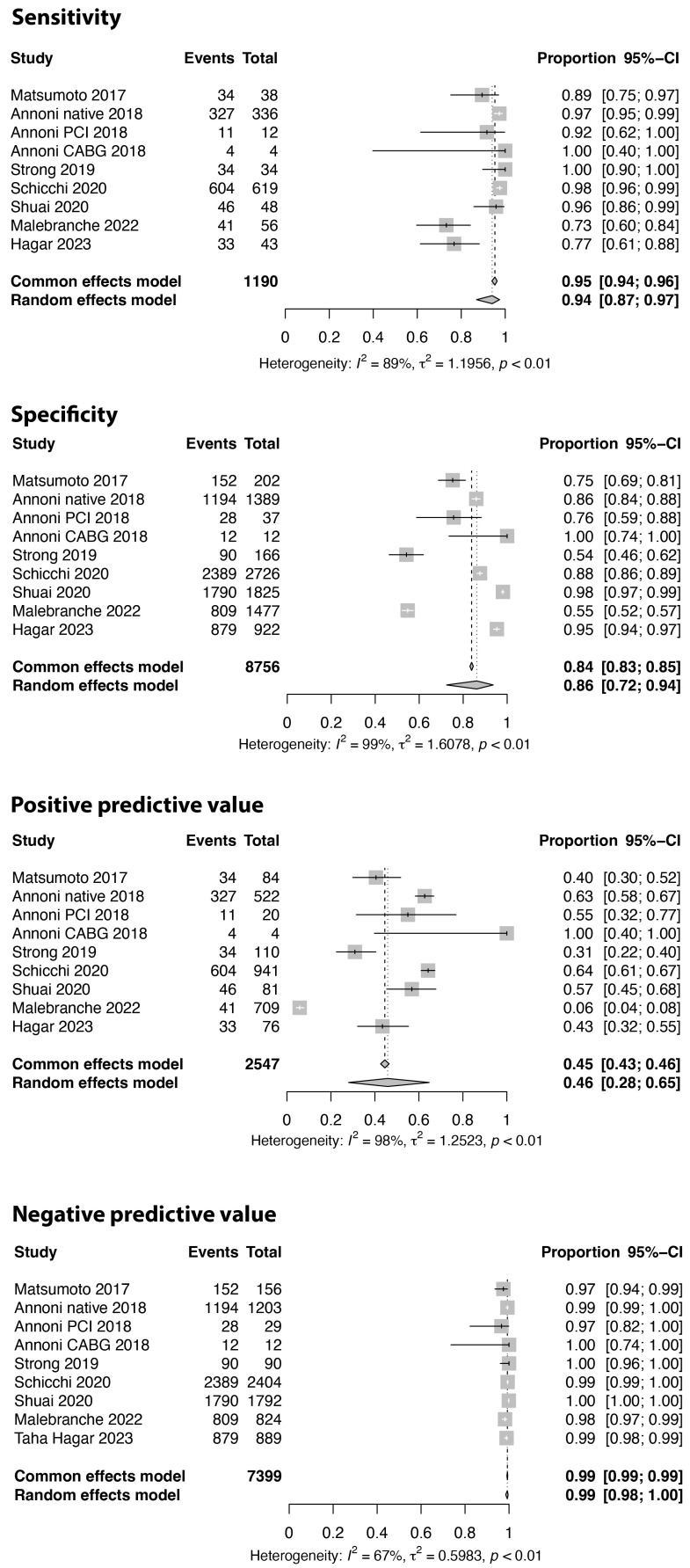
Sensitivity, specificity, positive and negative predictive values on the segment, coronary artery, level. Non-evaluable segments are considered as diseased/stenosed. Comparison between coronary computed tomography and invasive angiography, using a cut-off value of 50% lumen stenosis, are displayed [28,34,40,41,49,50,51].

Considering the unevaluable segments as diseased, nine studies with a total of 9946 segments were included. In the random effects meta-analysis, the sensitivity, specificity, PPV, and NPV were 0.94 (95% CI: 0.87–0.97), 0.86 (95% CI: 0.72–0.94), 0.46 (95% CI: 0.28–0.65), and 0.99 (95% CI: 0.98–1.00), respectively, in this analysis (Figure 9). As illustrated in Figure 9, a considerable level of variability in the values for specificity and PPV was seen between the included studies.

### 3.3. Analysis of Proximal Coronary Segments and Bypass Grafts

**Figure 10:**
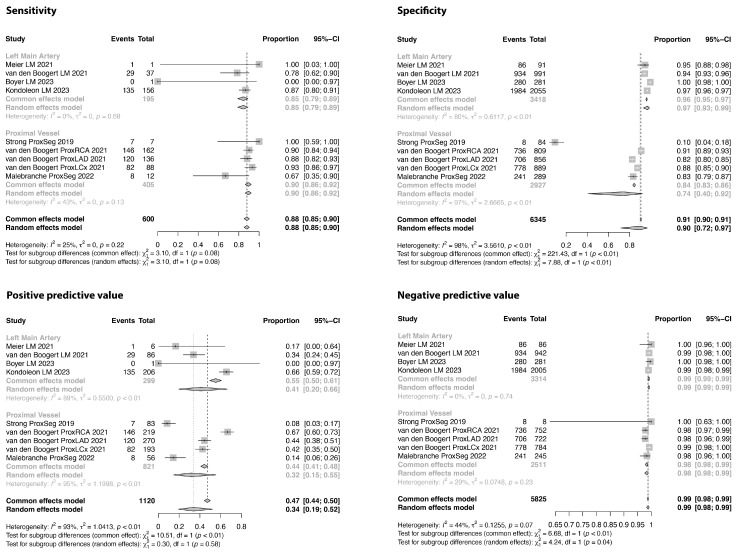
Sensitivity, specificity, positive and negative predictive values of the proximal coronary artery segments. Comparison between coronary computed tomography and invasive angiography, using a cut-off value of 50% lumen stenosis [29,38,40,42,51,52].

For this sub-analysis, we included 10 studies, reporting specifically on 7251 proximal evaluable segments. Figure 10 shows the results of the combined analysis, as well as when stratifying segments into left main coronary artery segments and other proximal segments. In the random effects meta-analysis, the sensitivity, specificity, PPV, and NPV were 0.88 (95% CI: 0.85–0.90), 0.90 (95% CI: 0.72–0.97), 0.34 (95% CI: 0.19–0.52), and 0.99 (95% CI: 0.98–0.99), respectively. The specificity and NPV for the left main coronary artery were 0.97 and 0.99 in this analysis. 

As many TAVI patients have undergone previous coronary artery bypass surgery, the diagnostic accuracy of CCTA in this context is of interest. Numerous studies excluded patients with previous CABG, explicitly. However, we were able to identify eight studies, including 2081 bypass segments, for the meta-analytic analysis. The sensitivity, specificity, PPV, and NPV were 0.88 (95% CI: 0.84–0.91), 0.97 (95% CI: 0.95–0.98), 0.82 (95% CI: 0.75–0.88), and 0.98 (95% CI: 0.97–0.99) in the random effects meta-analysis, respectively, (Figure 10).

## 4. Discussion

The current meta-analysis underscores the diagnostic accuracy of CCTA in patients undergoing TAVI and supports the use of this technology for the comprehensive assessment of patients with severe aortic stenosis awaiting percutaneous aortic valve interventions. The sensitivity and the negative predictive valueof CCTA in this setting are excellent and should allow for the exclusion of the vast majority of patients with CAD. In contrast, the positive predictive value and specificity are limited, and the number of false positive findings remains considerable. This is not surprising given the high calcific coronary burden of patients undergoing TAVI [54,55]. This is due to well-recognized blooming effects and artifacts that limit the evaluability of coronary segments [20]. 

Our results align with prior systematic reviews, expanding on the number of patients included, exploring additional patient-related factors, and employing a meta-regression approach. Gatti conducted a systematic review and meta-analysis involving 14 studies with 2533 patients. They found that CCTA has a high sensitivity (97%) and a moderate specificity (68%) for detecting obstructive CAD in TAVI patients. The positive and negative likelihood ratios were 3.0 and 0.05, respectively, with a diagnostic odds ratio of 60. The area under the hierarchical summary ROC curve was 0.96, indicating good diagnostic accuracy. The study also found that single heartbeat CT scanners had higher specificity compared to other scanners [18]. In 2018, including data synthesized from seven studies (with a total of 1275 patients), van den Boogert also concluded that CCTA had patient-based pooled sensitivity, specificity, PPV, and NPV values of 95%, 65%, 71%, and 94%, respectively. The study authors concluded that CCTA offers acceptable diagnostic accuracy for excluding significant coronary artery disease in many TAVI patients, potentially reducing the need for additional coronary angiographies by 37% in this high-risk group [19]. Not confined to TAVI, Chaikriangkrai examined the diagnostic accuracy of CCTA for CAD before surgical or percutaneous aortic valve replacement/implantation [21]. The authors included 13 studies with a total of 1498 patients. The analysis revealed a summary area under the curve of 0.96. The combined sensitivity, specificity, positive-likelihood ratio, and negative-likelihood ratio of CCTA in detecting substantial stenosis as evaluated by ICA were reported as 95%, 79%, 4.48, and 0.06, respectively. In the subgroup analysis, the diagnostic characteristics of CCTA were similar across surgical and transcatheter AVR. It was concluded that despite the high occurrence of CAD (43%) in patients with aortic stenosis, CCTA is an appropriate diagnostic tool with a reliable accuracy profile for determining the need for ICA. 

Compared to the previous meta-analytic reports, our study, with data from over 7500 patients, provides similar sensitivity, specificity, PPV, and NPV values of 95%, 73%, 64%, and 97%, respectively, on a per patient basis compared to previous reports, while also providing pooled estimates on a segmental, coronary artery, level. In contrast to the previous studies, our results particularly highlight the dependance of diagnostic accuracy on CAD prevalence in the population studied. This is in line with the other literature on CCTA not related to pre-TAVI assessment [56]. Our meta-analysis data also suggest that when using state-of-the-art single heartbeat scanners, the diagnostic accuracy may not be relevantly influenced by atrial fibrillation. Given the trade-off between false negatives and false positives, the choice of the underlying population and the appropriate use (if any) of pre-screening tools before CCTA remains a clinical challenge. Possible screening tools, affecting the pre-test probability of CAD, could be general CAD scoring systems [57,58]. or the degree of coronary calcification in the calcium scoring CT [40,59]. In addition, including or excluding patients with known CAD or previous coronary interventions, can affect the pre-test probability of CAD. Depending on the clinical preference, such efforts might be implemented to minimize the number of false positives (i.e., unnecessary ICAs in the setting of positive CCTA) or false negatives (missed CAD on CCTA). Our analysis illustrates that the choice of the underlying population affects the PPV and specificity in this setting. While it might be argued that the aim of CCTA must be to reduce the number of false negative individuals to avoid TAVI-related coronary complications, the immediate clinical value of proactive detection of coronary artery stenosis before TAVI, irrespective of the image modality employed, has been called into question recently [60,61,62]. Emerging evidence suggests that patients do not necessarily benefit from proactive PCI before TAVI and the optimal time point for coronary interventions may indeed be after the valve procedure [62]. Nevertheless, current recommendations still advocate comprehensive coronary assessment before TAVI, and this has traditionally been the domain of ICA [2,3,4]. An alternative approach could focus on hemodynamically relevant stenoses of the left main stem or proximal coronary arteries, rather than attempting to assess all coronary segments via CCTA. Our analysis indicates that these segments can be assessed with acceptable diagnostic accuracy in the current era. This may represent a clinically reasonable strategy, which should be considered based on local heart team discussion and operator preferences. Furthermore, the high concordance of CCTA and ICA in excluding patients with relevant left main artery stenosis is consistent with data from the ISCHEMIA trial. Analyzing data from the trial, Mancini et al. showed a 97.1% agreement between the methods for ruling out left main stenosis >50% in 1728 patients with a high likelihood of CAD [63].

### 4.1. Potential and Feasibility of CCTA

Based on the published literature and four meta-analyses, CCTA can be considered a reliable method to exclude obstructive CAD before aortic intervention, especially in patients with a low pre-test probability of CAD. From our interpretation of the data, the use of CCTA can potentially avoid the need for downstream ICA in at least 25–50% of the cases, reducing the risks and costs associated with invasive procedures. Pre-TAVI CCTA is feasible across a wide spectrum of patients, including those with atrial fibrillation and patients with previous coronary intervention or bypass surgery. Coronary bypass grafts, in particular, can be imaged with high diagnostic accuracy, as demonstrated by the current study. While still in the early stages, we believe that emerging technologies like photon counting CTs have great potential to enhance diagnostic accuracy further and decrease the necessity for downstream invasive assessment. Using this new technology, Hagar et al. (2023) recently published their experience using a dual-source photon-counting CT scanner (2 × 144 acquired slices; NAEOTOM Alpha^®^, Siemens Healthineers, Erlangen, Germany), with a retrospective electrocardiography-gated ultra-high-resolution scanning protocol. Assessing the results presented in Figure 2, the PPV of the scanner appears superior to that reported in studies in the corresponding time period. Therefore, given the risk profile of the included population, our results support the conclusion in the authors’ study, suggesting that this new technology shows highly promising diagnostic accuracy, despite the inclusion of a high-risk population with pronounced coronary calcification or prior PCI [34].

### 4.2. Limitations in Regard to the Evidence

The published studies cover more than a decade of CCTA experience, and remain heterogenous in terms of the inclusion criteria, frequency of CAD, scanner technology, and scanning protocols. Therefore, the results of different studies are partially inconclusive, due to the largely retrospective single-center nature of the studies with a limited sample size. Additionally, the lack of blinding of the CTA readers to the ICA results in some studies might introduce bias. With the increasing availability of single heartbeat scanner technology, issues such as motion artifacts or supraventricular arrhythmias appear increasingly manageable. However, limitations due to inconclusive results resulting from extensive local coronary calcification and the potential for false negatives remain an issue. Future research should focus on improving CCTA’s diagnostic performance and exploring its role in broader patient populations.

### 4.3. Limitations of the Current Analysis

While efforts were made to include all relevant studies and to assemble as much raw information as possible from individual studies, we cannot exclude the possibility that relevant studies might have been missed, or additional data might have been available. The results of the current study are, however, consistent with previous reports suggesting that this issue is likely to be of limited importance. Studies are heterogenous with regard to the CAD criteria, coronary segment models employed and, especially, local protocols or available CT scanner technology. This should be considered as it will increase heterogeneity and limit the generalizability of the results. We employed meta-regression analyses, which are recognized to be prone to ecological fallacy, where associations observed at the study level might not hold true at the individual level. Thus, the inferences made based on aggregate study data may not fully apply on an individual subject level. Further prospective studies with consistent protocols and scanner setups, utilizing state-of-the art CTs and standardized patient selection protocols are required to clarify the diagnostic accuracy of CCTA in the current era. Additionally, pooling raw data from published data, similar to the approach by van den Boogert et al. [52] or the collaborative meta-analysis of cardiac CT consortium [64] might be considered across the spectrum of pre-TAVI CCTA. 

### 4.4. Conclusions and Clinical Implications

The current study summarizes the current published evidence and is consistent with previous reports suggesting that CCTA can be a useful first-line test in the pre-TAVI workup. Concerns remain regarding patient selection, particularly in patients with severe coronary calcification. Overall, CCTA demonstrates an acceptable level of diagnostic accuracy in assessing obstructive CAD in patients referred for TAVI. Its role in reducing the need for invasive angiography may be significant, particularly in well-defined patient subgroups. In experienced hands, CCTA may obviate the need for approximately half of pre-TAVI ICAs, especially if only proximal and hemodynamically relevant lesions are of interest. However, limitations such as inconclusive results due to calcification and the potential for false negatives need to be considered. Due to the heterogenous approaches reported in the literature, centers need to develop, and ideally validate, their individual protocols to fit the local patient spectrum and clinical expectations. Future research should focus on improving CCTA’s diagnostic performance by incorporating novel CT scanner technology, harmonizing patient selection, and standardizing scanning protocols.

## Figures and Tables

**Table 1:** Overview of the characteristics of the included studies in chronological order.

Study	Year	Patients	Age	Males (%)	BMI	D.M.	AF	Hyperchol.	HT	PCI	CABG	Betablocker	CT Slices
Pontone	2011	60	80.0 ± 8.0	63.3%	25.0 ± 5.0	13.0%	0.0%	40.0%	67.0%	24.0%	16.0%	ivabradine	64
Andreini	2014	325	81.1 ± 6.6	40.6%	25.6 ± 4.4	30.0%	0.0%	53.0%	74.0%	15.1%	12.9%	ivabradine	64
Harris	2014	100	79.6 ± 9.9	61.0%	-	24.0%	36.0%	72.0%	92.0%	16.0%	41.0%	no	128
Hamdan	2015	115	80.4	43.6%	26.8	30.4%	7.8%	70.4%	85.2%	69.6%	20.0%	yes	256
Matsumoto	2017	60	84.4 ± 4.6	28.3%	22.3 ± 3.6	-	-	-	-	10.0%	3.3%	-	320
Rossi	2017	140	82.3 ± 7.7	48.6%	27.1 ± 5.3	21.0%	31.0%	59.0%	75.0%	0.0%	0.0%	no	128
Annoni	2018	115	82.5 ± 6.2	55.7%	26.7 ± 3.6	18.3%	13.0%	68.7%	71.3%	14.8%	13.9%	no	256
Hachulla	2019	84	84.8	47.6%	26.9	-	-	-	-	-	-	no	128
Strong	2019	200	83.4 ± 5.9	40.0%	26.6 ± 4.7	28.0%	33.5%	73.5%	92.5%	0.0%	0.0%	no	64
Schicchi	2020	223	79.2 ± 4.9	-	-	-	19.7%	-	-	35.0%	16.6%	no	192
Shuai	2020	121	73.3 ± 6.4	47.1%	22.6 ± 3.9	26.4%	27.2%	12.0%	37.1%	0.0%	0.0%	no	256
Meier	2021	127	82.3 ± 7.3	38.6%	26.5 ± 5.1	36.0%	-	54.3%	77.2%	16.5%	0.0%	no	64
Opolski	2021	475	82.6 ± 6.0	41.0%	27.5 ± 5,1	32.0%	19.0%	48.0%	95.0%	48.0%	19.0%	no	64
van den Boogert *	2021	1060	81.7 ± 6.6	51.4%	26.8 ± 4.9	21.3%	15.5%	51.8%	84.0%	29.8%	16.1%	yes	various
Bradt	2022	95	78.6 ± 8.8	47.4%	28.2 ± 6.6	30.5%	35.8%	74.7%	96.8%	9.4%	0.0%	yes	128
Gohmann	2022	460	79.6 ± 7.4	57.0%	29.4	-	-	-	-	0.0%	0.0%	no	128
Malebranche	2022	100	82.3 ± 6.5	30.0%	25.5 ± 5.6	20.0%	14.0%	-	84.0%	0.0%	0.0%	no	128
Peper	2022	338	81.0 ± 6.5	42.3%	26.6 ± 5.0	25.4%	-	29.3%	71.3%	0.0%	0.0%	yes	64 and 256
Zhang	2022	88	74.0 ± 6.0	56.8%	22.4 ± 4.1	9.1%	100.0%	-	27.3%	0.0%	0.0%	no	256
Boyer	2023	282	82.1 ± 7.2	43.3%	26.6 ± 5.1	28.7%	28.4%	39.0%	70.9%	0.0%	0.0%	yes	256
Hagar	2023	68	81.0 ± 7.0	47.1%	26.6 ± 4.5	22.0%	-	63.0%	82.0%	22.0%	1.0%	no	288
Khan	2023	192	82.0 ± 6.0	61.0%	-	-	-	-	-	2.6%	21.0%	-	64
Kondoleon	2023	2211	79.2 ± 8.5	53.4%	29.0 ± 7.4	33.3%	39.3%	-	87.6%	0.0%	16.1%	-	max. 256
Lecomte	2023	206	80.6 ± 6.1	44.7%	26.7 ± 4.6	-	20.0%	-	-	0.0%	0.0%	no	256
Renker	2023	192	81.9	36.5%	26.8	26.6%	42.2%	25.0%	94.8%	0.0%	0.0%	no	64 and 192
Sasaki	2023	21	86.0 ± 4.0	38.0%	21.6 ± 3.1	38.1%	-	57.1%	95.2%	14.3%	0.0%	no	192

AF = atrial fibrillation. BMI = body mass index. CABG = coronary artery bypass surgery. CT = computed tomography. D.M. = diabetes mellitus. HT = arterial hypertension. Hyperchol. = hypercholesterolemia. PCI = percutaneous coronary intervention. * Study pooling proximal coronary artery segment data from Andreini, Hamdan, Opolski, and Rossi.

**Table 2:** Details of individual studies reporting 2 × 2 contingency data (on the patient level for 50% coronary artery stenosis) comparing coronary computed tomographic angiography with invasive coronary angiography.

Study	Year	N	TPs	TNs	FPs	FNs
Pontone	2011	60	23	30	4	3
Andreini	2014	325	87	207	21	10
Harris	2014	100	72	15	12	1
Hamdan	2015	115	47	48	18	2
Matsumoto	2017	60	22	21	15	2
Rossi	2017	140	53	45	37	5
Annoni	2018	115	22	80	12	1
Strong	2019	200	69	76	55	0
Schicchi	2020	223	44	158	20	1
Shuai	2020	121	28	81	11	1
Opolski	2021	475	265	76	129	5
van den Boogert *	2021	1060	296	536	217	11
Bradt	2022	95	27	47	18	3
Gohmann	2022	388	135	113	137	3
Malebranche	2022	100	30	8	62	0
Peper	2022	338	50	176	97	15
Zhang	2022	88	24	57	7	0
Boyer	2023	282	43	211	23	5
Hagar	2023	68	23	37	7	1
Khan	2023	192	21	142	26	3

TPs = true positives, TNs = true negatives, FPs = false positives, FNs = false negatives. * Study pooling proximal coronary artery segment data from Andreini, Hamdan, Opolski, and Rossi.

**Table 3:** Overview of the results of the univariable meta-regression analysis, assessing the association between the patient and the CT-related characteristics and sensitivity, specificity, positive predictive and negative predictive values. Parameters with a *p*-value < 0.10 are printed in bold.

Variable	Sens Estimate	Sens *p*-Value	Spec Estimate	Spec *p*-Value	PPV Estimate	PPV *p*-Value	NPV Estimate	NPV *p*-Value
Study Year	0.043	0.912	−0.772	0.572	**−2.616**	**0.001**	0.323	0.157
Frequency of >50% stenosis	**0.181**	**0.006**	**−0.800**	**0.013**	**0.473**	**0.029**	**−0.122**	**0.070**
Prev. PCI (0/1)	0.073	0.143	0.047	0.860	**0.309**	**0.046**	−0.008	0.841
Prev. CABG (0/1)	0.108	0.280	0.143	0.745	**0.635**	**0.007**	0.010	0.886
Atrial fibrillation (0/1)	0.049	0.315	0.085	0.752	0.033	0.845	0.035	0.328
Males (%)	0.020	0.898	1.023	0.062	0.633	0.120	0.083	0.341
Age (years)	−0.319	0.552	**−2.747**	**0.074**	−1.956	0.134	−0.403	0.167
BMI (kg/m^2^)	0.325	0.708	−3.672	0.141	−2.264	0.257	−0.048	0.923
Diab. Mel. (%)	0.025	0.928	−0.548	0.522	−0.297	0.672	0.053	0.746
Hypercholesterolaemia (%)	0.169	0.153	−0.251	0.353	0.134	0.553	0.011	0.852
Hypertension (%)	8.952	0.450	**−53.277**	**0.008**	−14.830	0.527	−6.165	0.239
Betablocker (0/1)	**−8.206**	**0.001**	13.269	0.177	3.300	0.687	−2.081	0.149
CT slices	−0.009	0.582	0.066	0.216	0.002	0.960	0.004	0.592
CT whole heart coverage (0/1)	−2.493	0.354	**20.937**	**0.020**	1.853	0.804	1.271	0.400

BMI = body mass index, CT = computed tomography, Diab. Mel. = diabetes mellitus, PPV = positive predictive value, NPV = negative predictive value, Sens = sensitivity, Spec = specificity.

**Table 4:** Association between prevalence of coronary artery disease (defined as at least 50% lumen stenosis in invasive coronary angiography) and results of the coronary computed tomography angiography.

Prevalence	CT Suggests CAD–ICA Neg.	CT Suggests no CAD–ICA Pos.	CT Suggests CAD–ICA Confirmed	CT Suggests no CAD–ICA Confirmed	% Correct
5%	25.8% (95% CI: 17.7–35.9%)	0.2% (95% CI: 0.1–0.4%)	4.8% (95% CI: 4.6–4.9%)	69.2% (95% CI: 59.1–77.3%)	74.0%
10%	24.5% (95% CI: 16.8–34.0%)	0.5% (95% CI: 0.3–0.7%)	9.5% (95% CI: 9.3–9.7%)	65.5% (95% CI: 56.0–73.2%)	75.0%
20%	21.7% (95% CI: 14.9–30.2%)	0.9% (95% CI: 0.6–1.5%)	19.1% (95% CI: 18.5–19.4%)	58.3% (95% CI: 49.8–65.1%)	77.4%
30%	19.0% (95% CI: 13.0–26.5%)	1.4% (95% CI: 0.9–2.2%)	28.6% (95% CI: 27.8–29.1%)	51.0% (95% CI: 43.5–57.0%)	79.6%
40%	16.3% (95% CI: 11.2–22.7%)	1.8% (95% CI: 1.1–2.9%)	38.2% (95% CI: 37.1–38.9%)	43.7% (95% CI: 37.3–48.8%)	81.9%
50%	13.6% (95% CI: 9.3–18.9%)	2.3% (95% CI: 1.4–3.7%)	47.7% (95% CI: 46.3–48.6%)	36.4% (95% CI: 31.1–40.7%)	84.1%
60%	10.9% (95% CI: 7.5–15.1%)	2.8% (95% CI: 1.7–4.4%)	57.2% (95% CI: 55.6–58.3%)	29.1% (95% CI: 24.9–32.5%)	86.3%
70%	8.2% (95% CI: 5.6–11.3%)	3.2% (95% CI: 2–5.1%)	66.8% (95% CI: 64.9–68%)	21.8% (95% CI: 18.7–24.4%)	88.6%
	false positives	false negatives	true positives	true negatives	

CAD = coronary artery disease, CT = computed tomography, ICA = invasive coronary angiography. The prevalence of 30% corresponds to the average rate estimated in the current study (green color = accurate diagnosis, red color = inaccurate diagnosis).

## Data Availability

Raw data included in the current study will be shared upon reasonable request to the corresponding author.

## Supplementary Materials

The following supporting information can be downloaded at: https://www.mdpi.com/article/10.3390/jcm13020631/s1. Reference [65] is cited in the Supplementary Materials.
