# Supplementary material for: Utility of Coronary Computed Tomography Angiography in Patients Undergoing Transcatheter Aortic Valve Implantation: A Meta-Analysis and Meta-Regression Based on Published Data from 7458 Patients
Source: J Clin Med. 2024 Jan 22;13(2):631. doi: 10.3390/jcm13020631 (PMC10816478; doi:10.3390/jcm13020631)
Supplement: Supplementary file 1 [file jcm-13-00631-s001.zip › jcm-2823398-supplementary.pdf]

Supplementary Material:

Results of the Funnel plot test for diagnostic odds ratios (Deeks et al. 2005)[1] providing no evidence of relevant publication bias.

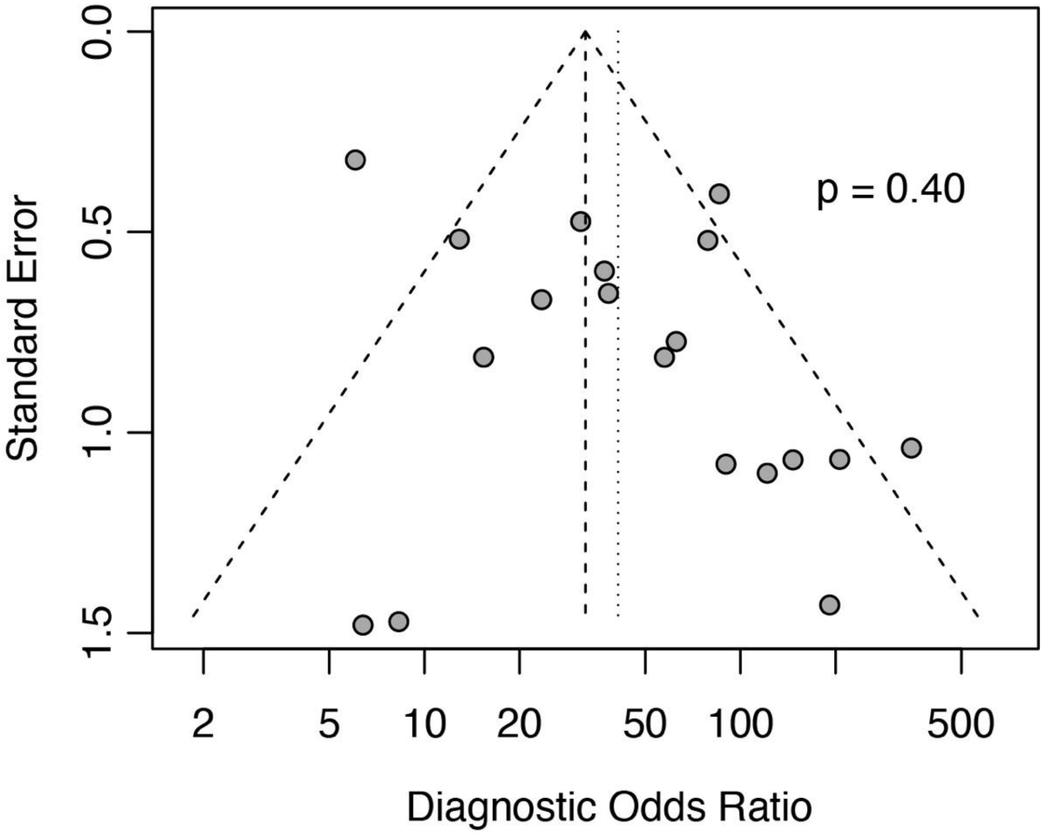

Descriptive information on inclusion and exclusion criteria by original study:

Andreini 2014[2]

Inclusion Criteria:
Patients undergoing invasive coronary angiography (ICA) and cardiovascular multi-detector computed tomography (MDCT) within a transcatheter aortic valve replacement screening protocol were considered for enrollment in the study.
In all patients, ICA was performed approximately 3 ± 2 days after MDCT.

Exclusion Criteria:
Hypersensitivity to contrast agents.
Impaired renal function (creatinine clearance below 60 mL/min).
Inability to sustain a 15-second breath hold.
Cardiac arrhythmias.

Annoni 2018[3]

Inclusion Criteria:
Patients were referred to the hospital for CT and invasive coronary angiography (ICA) before transcatheter aortic valve implantation planning.
The patients had severe symptomatic aortic valve stenosis and were considered at high surgical risk.
In all patients, ICA was performed at least 3 days after the CT scan.

Exclusion Criteria:
Previous severe adverse reactions to an iodinated contrast agent.
Body mass index (BMI) of 38 kg/m² or higher (due to the need for a different scan protocol).
Severely impaired renal function with an estimated glomerular filtration rate (eGFR) of less than 30 ml/min/1.73 m².

Boyer 2023[4]

Inclusion Criteria:
All consecutive adult patients referred to the center for transcatheter aortic valve implantation (TAVI) due to severe symptomatic aortic stenosis were enrolled.
The study included data collected in the France TAVI registry.
As part of the standard clinical practice, all patients underwent pre-interventional coronary computed tomography angiography (CCTA) to assess valve morphology and invasive coronary angiography (ICA) for coronary artery disease (CAD) evaluation.

Exclusion Criteria:
Patients with a prior history of percutaneous coronary intervention (PCI) or coronary artery bypass grafting (CABG) were not included.
Patients with incomplete CCTA or those who underwent PCI before CCTA were excluded.

Brandt 2022[5]

Inclusion Criteria:
Consecutive patients with symptomatic and severe aortic stenosis (AS).
Patients underwent coronary computed tomography angiography (CCTA) followed by invasive coronary angiography (ICA) with quantitative coronary angiography (QCA) within 3 months.
The procedures were part of the pre-procedural diagnostic workup before transcatheter aortic valve replacement (TAVR) conducted between September 2014 and December 2019.

Exclusion Criteria:
Insufficient image quality for the visual evaluation of the coronary arteries or the computation of CT-derived fractional flow reserve (CT-FFR).
History of previous coronary artery bypass graft (CABG) and/or percutaneous coronary intervention (PCI).
Presence of chronic total occlusions

Chava 2017[6]

Inclusion Criteria:
Patients who were candidates for transcatheter aortic valve replacement (TAVR) and were under the age of 75 years.
Patients who did not have pulmonary edema.
Patients whose heart rate after beta blocker administration was below 70 beats per minute.
Patients who had no prior stenting of proximal coronary arteries.
The protocol for proceeding with CT angiogram was followed as per institutional practice.

Gohmann 2022[7]

Inclusion Criteria:
Consecutive patients referred for pre-transcatheter aortic valve replacement (TAVR) CT evaluation.
Patients routinely underwent invasive coronary angiography (ICA), which was omitted in select cases where obstructive coronary artery disease (CAD) could be effectively ruled out on coronary computed tomography angiography (cCTA).

Exclusion Criteria:
Patients not undergoing retrospectively electrocardiography (ECG)-gated CT prior to coronary artery bypass grafting.
Patients were also excluded if their cCTA was positive for obstructive CAD (CAD+) but had not undergone ICA within 3 months of the CT examination.
Exclusion also applied if ICA was not available or not suitable for quantitative assessment of the coronary arteries (QCA)

Hachulla 2019[8]

Inclusion Criteria:
Consecutive, adult patients with severe aortic valve stenosis eligible for TAVI between 01/2014 and 08/2015

Hagar 2023[9]

Inclusion Criteria:
Patients with severe aortic valve stenosis.
Patients who were referred for pre-transcatheter aortic valve replacement (TAVR) work-up.
Severe aortic valve stenosis had to be confirmed with transthoracic echocardiography.
There should be no contraindication to iodinated contrast-enhanced CT.
Diagnostic invasive coronary angiography (ICA) was performed within 30 days of the CT.

Hamdan 2015[10]

Inclusion Criteria:
Consecutive patients referred for TAVI. All patients underwent preprocedural CT angiography and invasive X-ray coronary
angiography.

Exclusion Criteria:
Long delay (>1 year) between CT and invasive coronary angiography
Inability to hold the breath.
Left ventricular assist devices.

Harris 2014[11]

Inclusion Criteria
Consecutive patients undergoing TAVI CT and angiographic studies within 3 months.

Kondoleon 2023[12]

Inclusion Criteria:
Patients referred to the Cleveland Clinic for TAVI January 2015 to November 2021
All patients with a preprocedure CTA, preprocedure ICA, and without prior percutaneous intervention (PCI) were included in the study.

Khan 2023[13]

Inclusion Criteria:
Consecutive patients referred for TAVI. All patient underwent coronary angiography. CT scans blind-reported.

Lecompte 2023[14]

Inclusion Criteria
Registry Participation: All patients were part of the France-TAVI registry.
Consent for Data Use: Agreed to have their data used for research purposes.
Underwent TAVI-CT and Coronary Angiography: Patients who underwent TAVI-CT (transcatheter aortic valve implantation computed tomography) and coronary angiography before TAVI (transcatheter aortic valve implantation) at the study's institution.
Time Frame: Inclusion of patients from December 2021 to July 2022.

Exclusion Criteria
Coronary Stents or Bypass Grafts on TAVI-CT: Patients with coronary stents or coronary bypass grafts evident in TAVI-CT examinations.

Malebranche 2022[15]

Inclusion Criteria
Patients undergoing TAVI at Bern University Hospital, Switzerland.
Enrolment into a prospective institutional registry that is part of the Swiss TAVI registry.
Patients with symptomatic severe aortic stenosis undergoing CT angiography as part of the routine pre-procedural TAVI work-up starting from June 2018.
Independent blinded readers assessed CTA with regard to the coronary arteries and performed quantitative coronary angiography (QCA).

Exclusion Criteria
Prior percutaneous coronary intervention or coronary artery bypass grafting.
Previous pacemaker implantation or valve-in-valve intervention.
CT angiographies (CTAs) performed at external institutions.

Matsumoto 2017[16]

Inclusion Criteria
Patients with severe symptomatic aortic stenosis.
Patients receiving TAVI planning CT / undergoing TAVI between April 4, 2014, and March 31, 2015.
Participants with CT and invasive coronary angiography (CAG) within 2 months before the TAVI procedure.
Use of a balloon-expandable Edwards SAPIEN XT heart valve for the TAVI procedure.
Approval by the institutional review board and written informed consent from each participant.

Exclusion Criteria
Patients scanned with protocols different from the Variable Helical Pitch (VHP) scanning protocol.
Non-adherence to the VHP scanning protocol.

Meier 2021[17]

Inclusion Criteria
Patients undergoing transcatheter aortic valve implantation (TAVI) from June 1, 2013, to December 31, 2017.
Part of the SWISS TAVI registry, a national multi-center prospective cohort study in Switzerland.
All patients evaluated for potential TAVI in the center were eligible.
Ethical approval was obtained, and all patients provided written informed consent.

Exclusion Criteria
Previous coronary artery bypass grafting (CABG).
Unavailability of computed tomography angiography (CTA) images.

Opolski 2015[18]

Inclusion Criteria
Patients with severe aortic stenosis referred for standard diagnostic work-up between January 2011 and November 2013.
Those who had both CTA and ICA data available.
CTA was performed within 4 weeks of ICA.
Patients with arrhythmias, including atrial fibrillation and pacemaker-dependent rhythm, were included.
No additional medications for heart rate control or vasodilation administered before the CTA scan.
Approval by the institutional ethics committee and written informed consent from all participants.

Exclusion Criteria
Patients excluded due to potentially preventable technical or human-related factors during the CTA examination. This included significant patient movements or contrast extravasation, resulting in significant motion artifacts in 21 cases (4%) and poor contrast opacification in 14 cases (3%).
A total of 35 patients (7%) were excluded from the final analysis due to these reasons.
Pepper 2022[19]

Inclusion Criteria
Consecutive patients with severe symptomatic aortic valve stenosis undergoing TAVR work-up between 2015 and 2019.

All patients underwent CCTA and ICA within 3 months.

Exclusion Criteria
Previous coronary intervention or surgery, affecting CT imaging and CT-FFR computation.
Unavailability of CCTA images, incompatibility with CT-FFR software, vessels not traceable due to poor imaging quality or artifacts, aberrant anatomy, and stenosis at the ostium.

Pontone 2011[20]

Inclusion Criteria:
Consecutive patients with aortic stenosis referred for transcatheter aortic valve implantation (TAVI) between January 2008 and June 2009.

Exclusion Criteria:
Impaired renal function (creatinine clearance below 30 mL/min).
Inability to sustain a 10-second breathhold.
Atrial fibrillation.
Other arrhythmias.
Patients who did not achieve a target heart rate below 70 beats/min after administration of ivabradine.

Rossi 2017[21]

Inclusion Criteria
Consecutive patients screened for the study.
Those with severe aortic stenosis undergoing evaluation for TAVR.
Patients who underwent computed tomography angiography (CTA) and invasive coronary angiography (ICA) as part of their diagnostic work-up.

Exclusion Criteria
Patients with a history of previous revascularization (110 patients excluded).
Those with missing or incomplete ICA images (89 patients excluded), leading to the exclusion of 199 patients in total

Renker 2023[22]

Inclusion Criteria
Patients with symptomatic severe AS who underwent echocardiography, CT, and ICA at one institution from June 2014 to April 2017.
Between June 2014 and February 2016 CT was performed using first-generation DSCT and between April 2016 and April 2017 using third-generation DSCT.

Exclusion criteria
CT data sets between February and April 2016.

patients with more than 3 months between CT and !CA were excluded
Patients with known CAD (highest risk category) or renal dysfunction estimated glomerular filtration rate< 30 mL/min/1. 73 m2 were excluded.

Sasaki 2023[23]

Inclusion Criteria
Adult patients with severe AS.
Patients with moderate or greater (30–90%) stenotic lesions on coronary computed tomography angiography (cCTA) and coronary angiography (CAG) prior to TAVR.
Patients with successful TAVR.
Exclusion Criteria
Patients with hemodynamic instability or major procedural complications.
Patients with prior left main trunk stenting, stenting in one or more coronary arteries, or prior coronary artery bypass grafting. Severe artifacts or poor contrast. Motion or misalignment artifact.

Schicchi 2020[24]

Inclusion Criteria
Consecutive patients referred for computed tomography (CT) and invasive coronary angiography (ICA) for TAVI planning between April 2018 and April 2019.
All patients had ICA performed at least 3 days after CT.

Exclusion Criteria
Patients with severe adverse reactions to an iodinated contrast agent (5 patients excluded).
Patients with an estimated glomerular filtration rate less than 30 ml/min/1.73m2 (8 patients excluded).

Shuai 2020[25]

Inclusion Criteria
Consecutive patients with severe symptomatic aortic valve stenosis.
Patients scheduled for TAVR planning CT between November 1, 2018, and November 30, 2019.

Exclusion Criteria
Known allergy to iodinated contrast agent.
Renal insufficiency or hyperthyroidism.
Previous coronary revascularization by percutaneous coronary intervention (PCI) and/or coronary artery bypass grafts (CABG).

Strong 2019[26]

Inclusion Criteria
Patients with severe symptomatic aortic stenosis.
Undergoing both pre-procedural CTA and invasive coronary angiography (ICA) between October 2015 and March 2018.
Considered potential candidates for TAVI based on estimated life expectancy greater than one year and intermediate or high surgical risk.
The TAVI screening protocol including CTA and ICA.

Exclusion Criteria
Candidates with a time interval between CTA and ICA greater than six months.
Patients with a previous history of acute coronary syndrome (ACS) and/or previous coronary revascularization (either percutaneous or surgical).
Patients with poor image quality were not excluded.

Zhang 2021[27]

Inclusion Criteria
Consecutive TAVI candidates with AF enrolled between June 2018 and June 2020.
TAVI planning computed tomography (CT) and invasive coronary catheter angiography (ICA) within 1 month after the CT examination.

Exclusion Criteria
Patients with previous coronary revascularization by percutaneous coronary intervention and/or coronary artery bypass grafts.

Figure S1. Sensitivity, Specificity, Positive and Negative Predictive Value on a patient level, comparing coronary computed tomography with invasive angiography using a cut-off value of 70% lumen stenosis.

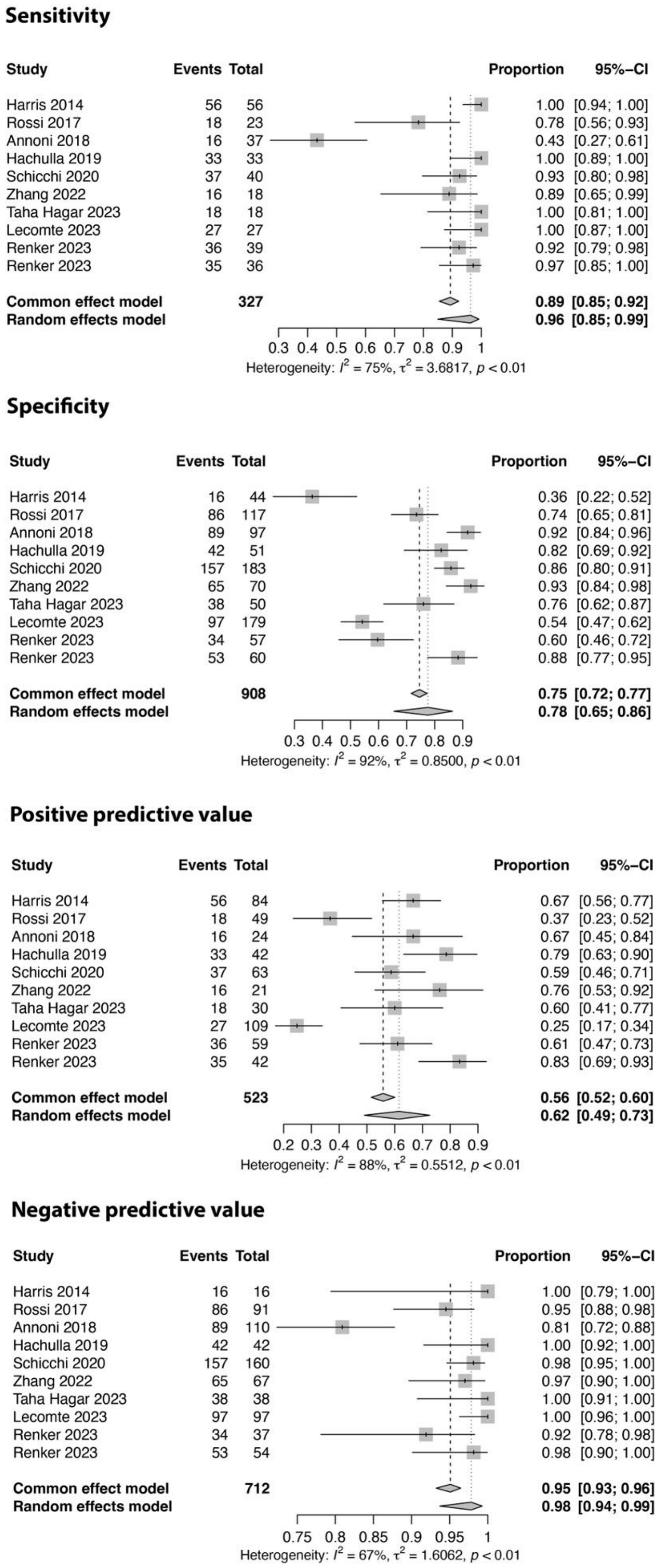

References

1. Deeks JJ, Macaskill P, Irwig L. The performance of tests of publication bias and other sample size effects in systematic reviews of diagnostic test accuracy was assessed. *J Clin Epidemiol*. 2005;58:882-893. doi: 10.1016/j.jclinepi.2005.01.016
2. Andreini D, Pontone G, Mushtaq S, Bartorelli AL, Ballerini G, Bertella E, Segurini C, Conte E, Annoni A, Baggiano A, et al. Diagnostic accuracy of multidetector computed tomography coronary angiography in 325 consecutive patients referred for transcatheter aortic valve replacement. *Am Heart J*. 2014;168:332-339. doi: 10.1016/j.ahj.2014.04.022
3. Annoni AD, Andreini D, Pontone G, Mancini ME, Formenti A, Mushtaq S, Baggiano A, Conte E, Guglielmo M, Muscogiuri G, et al. CT angiography prior to TAVI procedure using third-generation scanner with wide volume coverage: feasibility, renal safety and diagnostic accuracy for coronary tree. *Br J Radiol*. 2018;91:20180196. doi: 10.1259/bjr.20180196
4. Boyer J, Bartoli A, Deharo P, Vaillier A, Ferrara J, Barral PA, Jaussaud N, Morera P, Porto A, Collart F, et al. Feasibility of Non-Invasive Coronary Artery Disease Screening with Coronary CT Angiography before Transcatheter Aortic Valve Implantation. *J Clin Med*. 2023;12. doi: 10.3390/jcm12062285
5. Brandt V, Schoepf UJ, Aquino GJ, Bekeredjian R, Varga-Szemes A, Emrich T, Bayer RR, 2nd, Schwarz F, Kroencke TJ, Tesche C, et al. Impact of machine-learning-based coronary computed tomography angiography-derived fractional flow reserve on decision-making in patients with severe aortic stenosis undergoing transcatheter aortic valve replacement. *Eur Radiol*. 2022;32:6008-6016. doi: 10.1007/s00330-022-08758-8
6. Chava S, Gentchos G, Abernethy A, Leavitt B, Terrien E, Dauerman HL. Routine CT angiography to detect severe coronary artery disease prior to transcatheter aortic valve replacement. *J Thromb Thrombolysis*. 2017;44:154-160. doi: 10.1007/s11239-017-1521-1
7. Gohmann RF, Pawelka K, Seitz P, Majunke N, Heiser L, Renatus K, Desch S, Lauten P, Holzhey D, Noack T, et al. Combined cCTA and TAVR Planning for Ruling Out Significant CAD: Added Value of ML-Based CT-FFR. *JACC Cardiovasc Imaging*. 2022;15:476-486. doi: 10.1016/j.jcmg.2021.09.013
8. Hachulla AL, Noble S, Ronot M, Guglielmi G, de Perrot T, Montet X, Vallee JP. Low Iodine Contrast Injection for CT Acquisition Prior to Transcatheter Aortic Valve Replacement: Aorta Assessment and Screening for Coronary Artery Disease. *Acad Radiol*. 2019;26:e150-e160. doi: 10.1016/j.acra.2018.06.016
9. Hagar MT, Soschynski M, Saffar R, Rau A, Taron J, Weiss J, Stein T, Faby S, von Zur Muehlen C, Ruile P, et al. Accuracy of Ultrahigh-Resolution Photon-counting CT for Detecting Coronary Artery Disease in a High-Risk Population. *Radiology*. 2023;307:e223305. doi: 10.1148/radiol.223305
10. Hamdan A, Wellnhofer E, Konen E, Kelle S, Goitein O, Andrada B, Raanani E, Segev A, Barbash I, Klempfner R, et al. Coronary CT angiography for the detection of coronary

artery stenosis in patients referred for transcatheter aortic valve replacement. *J Cardiovasc Comput Tomogr*. 2015;9:31-41. doi: 10.1016/j.jcct.2014.11.008
11. Harris BS, De Cecco CN, Schoepf UJ, Steinberg DH, Bayer RR, Krazinski AW, Dyer KT, Sandhu MK, Zile MR, Meinel FG. Dual-source CT imaging to plan transcatheter aortic valve replacement: accuracy for diagnosis of obstructive coronary artery disease. *Radiology*. 2015;275:80-88. doi: 10.1148/radiol.14140763
12. Kondoleon NP, Layoun H, Spilias N, Sipko J, Kanaan C, Harb S, Reed G, Puri R, Yun J, Krishnaswamy A, et al. Effectiveness of Pre-TAVR CTA as a Screening Tool for Significant CAD Before TAVR. *JACC Cardiovasc Interv*. 2023;16:1990-2000. doi: 10.1016/j.jcin.2023.05.030
13. Khan S, Gedela S, Frost OMT, Galinato M, Crawford I, Smith S, Fawaz S, Simpson R, Singh A, Karamasis GV, et al. Noncoronary Gated Transcatheter Aortic Valve Replacement Computed Tomography Scans Can Safely Replace Invasive Coronary Angiography Pre-Transcatheter Aortic Valve Replacement. *Struct Heart*. 2023;7:100123. doi: 10.1016/j.shj.2022.100123
14. Lecomte A, Serrand A, Marteau L, Carlier B, Manigold T, Letocart V, Warin Fresse K, Nguyen JM, Serfaty JM. Coronary artery assessment on pre transcatheter aortic valve implantation computed tomography may avoid the need for additional coronary angiography. *Diagn Interv Imaging*. 2023. doi: 10.1016/j.diii.2023.06.006
15. Malebranche D, Hoffner MKM, Huber AT, Cicovic A, Spano G, Bernhard B, Bartkowiak J, Okuno T, Lanz J, Raber L, et al. Diagnostic performance of quantitative coronary artery disease assessment using computed tomography in patients with aortic stenosis undergoing transcatheter aortic-valve implantation. *BMC Cardiovasc Disord*. 2022;22:178. doi: 10.1186/s12872-022-02623-8
16. Matsumoto S, Yamada Y, Hashimoto M, Okamura T, Yamada M, Yashima F, Hayashida K, Fukuda K, Jinzaki M. CT imaging before transcatheter aortic valve implantation (TAVI) using variable helical pitch scanning and its diagnostic performance for coronary artery disease. *Eur Radiol*. 2017;27:1963-1970. doi: 10.1007/s00330-016-4547-4
17. Meier D, Depierre A, Topolsky A, Roguelov C, Dupre M, Rubimbura V, Eeckhout E, Qanadli SD, Muller O, Mahendiran T, et al. Computed Tomography Angiography for the Diagnosis of Coronary Artery Disease Among Patients Undergoing Transcatheter Aortic Valve Implantation. *J Cardiovasc Transl Res*. 2021;14:894-901. doi: 10.1007/s12265-021-10099-8
18. Opolski MP, Kim WK, Liebetrau C, Walther C, Blumenstein J, Gaede L, Kempfert J, Van Linden A, Walther T, Hamm CW, et al. Diagnostic accuracy of computed tomography angiography for the detection of coronary artery disease in patients referred for transcatheter aortic valve implantation. *Clin Res Cardiol*. 2015;104:471-480. doi: 10.1007/s00392-014-0806-z
19. Peper J, Becker LM, van den Berg H, Bor WL, Brouwer J, Nijenhuis VJ, van Ginkel DJ, Rensing B, Ten Berg JM, Timmers L, et al. Diagnostic Performance of CCTA and CT-FFR for the Detection of CAD in TAVR Work-Up. *JACC Cardiovasc Interv*. 2022;15:1140-1149. doi: 10.1016/j.jcin.2022.03.025
20. Pontone G, Andreini D, Bartorelli AL, Annoni A, Mushtaq S, Bertella E, Formenti A, Cortinovis S, Alamanni F, Fusari M, et al. Feasibility and accuracy of a comprehensive multidetector computed tomography acquisition for patients referred for balloon-

expandable transcatheter aortic valve implantation. *Am Heart J*. 2011;161:1106-1113. doi: 10.1016/j.ahj.2011.03.003

21. Rossi A, De Cecco CN, Kennon SRO, Zou L, Meinel FG, Toscano W, Segreto S, Achenbach S, Hausleiter J, Schoepf UJ, et al. CT angiography to evaluate coronary artery disease and revascularization requirement before trans-catheter aortic valve replacement. *J Cardiovasc Comput Tomogr*. 2017;11:338-346. doi: 10.1016/j.jcct.2017.06.001
22. Renker M, Steinbach R, Schoepf UJ, Fischer-Rasokat U, Choi YH, Hamm CW, Rolf A, Kim WK. Comparison of First-generation and Third-generation Dual-source Computed Tomography for Detecting Coronary Artery Disease in Patients Evaluated for Transcatheter Aortic Valve Replacement. *J Thorac Imaging*. 2023;38:165-173. doi: 10.1097/RTI.0000000000000699
23. Sasaki S, Kawamori H, Toba T, Takeshige R, Fukuyama Y, Hiromasa T, Fujii H, Hamana T, Osumi Y, Iwane S, et al. Diagnostic Accuracy of Pre-Transcatheter Aortic Valve Replacement Nitroglycerin-Free Fractional Flow Reserve-Computed Tomography-Based Physiological Assessment in Patients With Severe Aortic Stenosis for Predicting Post-Transcatheter Aortic Valve Replacement Ischemia. *Circ J*. 2023. doi: 10.1253/circj.CJ-23-0312
24. Schicchi N, Fogante M, Pirani PE, Agliata G, Piva T, Tagliati C, Marcucci M, Francioso A, Giovagnoni A. Third generation dual source CT with ultra-high pitch protocol for TAVI planning and coronary tree assessment: feasibility, image quality and diagnostic performance. *Eur J Radiol*. 2020;122:108749. doi: 10.1016/j.ejrad.2019.108749
25. Shuai T, Li W, You Y, Deng L, Li J, Peng L, Li Z. Combined Coronary CT Angiography and Evaluation of Access Vessels for TAVR Patients in Free-Breathing with Single Contrast Medium Injection Using a 16-cm-Wide Detector CT. *Acad Radiol*. 2021;28:1662-1668. doi: 10.1016/j.acra.2020.08.003
26. Strong C, Ferreira A, Teles RC, Mendes G, Abecasis J, Cardoso G, Guerreiro S, Freitas P, Santos AC, Saraiva C, et al. Diagnostic accuracy of computed tomography angiography for the exclusion of coronary artery disease in candidates for transcatheter aortic valve implantation. *Sci Rep*. 2019;9:19942. doi: 10.1038/s41598-019-56519-3
27. Zhang Y, Li Z, You Y, Peng L, Li J, Shuai T. Image quality and diagnostic performance evaluation in transcatheter aortic valve implantation candidates with atrial fibrillation using a whole-heart coverage CT scanner. *Eur Radiol*. 2022;32:1034-1043. doi: 10.1007/s00330-021-08187-z
